# Post COVID-19 Reflections and Questions: How Prepared Are We for the Next Pandemic?

**DOI:** 10.3390/ijms25020859

**Published:** 2024-01-10

**Authors:** George J. Kontoghiorghes, Annita Kolnagou, Christina N. Kontoghiorghe

**Affiliations:** Postgraduate Research Institute of Science, Technology, Environment and Medicine, 3 Ammochostou Street, Limassol 3021, Cyprus

While the end of the COVID-19 pandemic was announced earlier in 2023 by WHO, the currently dominating COVID-19 virus variants, such as the omicron sub-lineages XBB.1.5, XXB.1.16 and EG.5, appear to be progressively causing similar numbers of infections and fatalities to those of influenza virus variants [1,2,3,4]. The current strategy against COVID-19 mainly involves protection through vaccination and, for selected categories of symptomatic SARS-CoV-2-infected patients, treatment with the antiviral drugs remdesivir, molnupiravir, and the combination of nirmatrevir with ritonavir (paxlovid) (Figure 1) [5,6,7,8]. In some cases, the neutralizing monoclonal antibody sotrovimab is also used to treat symptomatic SARS-CoV-2-infected patients [9,10].

In the meantime, and despite reduced interest from the mass media regarding the COVID-19 pandemic, many scientific and other questions still remain, involving all aspects of human activity, including pharmacological and other therapeutic approaches, the perception and implications of the disease, as well as future strategies for COVID-19 and other similar infectious diseases [11].

A major setback in the design of therapeutic strategies against COVID-19 was the absence of effective antiviral drugs at the earlier stages of the pandemic, which caused millions of fatalities. In this context, many lives of SARS-CoV-2-infected patients could have been saved, had the efficacy of current antiviral therapies involving for example the repurposed antiviral drugs remdesivir and molnupiravir been identified and made available sooner (Figure 1).

Other drawbacks of the therapeutic strategies and responses in relation to the COVID-19 pandemic include the lack of co-ordination among countries for the prevention of and therapy for the disease, the difference between poor and rich nations in terms of access to therapeutics, misunderstandings related to the diagnosis and transmission of the disease, e.g., the late decision to use adequate protection with masks and the adoption of the delayed treatment option of the “herd immunity” model in the UK, delays in the regulatory approval of emergency antiviral and other repurposed drugs, etc. [11,12,13].

Further complications have also been identified as affecting the treatment and the overall rate of morbidity and mortality of infected patients during the COVID-19 pandemic period. These include the insufficient information and education of the public on viral transmission and safety, tedious procedures for the diagnosis of infected patients, the insufficient introduction of special measures to protect more susceptible groups of the population such as older populations and other categories of affected patients, the pressure on health systems and inadequacy of hospitals for treating a large number of patients, the limited or lack of co-ordination/crosstalk between academic investigators as well as academic investigators and industry, etc. [11].

Despite the difficult circumstances during the COVID-19 pandemic, which affected the lives of everyone, the response of the scientific and medical community to work towards a rapid diagnosis and possible treatment was instant. In this context, thousands of targets have been identified, and many more therapeutic solutions have been proposed during the pandemic period for the prevention, diagnosis and treatment of all the different stages of COVID-19, caused by the SARS-CoV-2 virus. The pharmacological proposal to treat infected patients included in most cases new investigational drugs, repurposed drugs, nutraceuticals, different drug combinations and vaccines [14,15,16,17,18,19].

Different approaches were adopted regarding drug development and emergency testing or use in COVID-19 patients. In particular, the relatively short life cycle of SARS-CoV-2 and associated toxicity effects suggested that the proposed therapeutic drugs against the virus should have exerted their therapeutic activity to reduce mortality in a matter of a few days or weeks. In considering the risk/benefit assessment for this short time window, the therapeutic approach could, in general, allow for the administration of repeated effective doses of antiviral drugs, usually at the maximum dose of the regulatory approved range. Furthermore, this short treatment period also allowed for the rapid approval of drug trials due to the emergency, life-threatening COVID-19 pandemic conditions [13]. Similar approaches have also been considered for the development of drugs for the treatment of the severe acute respiratory syndrome and other life-threatening side effects of the virus, affecting other systems in addition to the respiratory system, such as the cardiovascular, gastrointestinal, nervous, immune and hematopoietic systems [20,21,22,23,24,25]. A different approach has been considered for the development of other drugs that are related to each of the different stages of COVID-19 and also for the “long COVID” side effects [11,26,27,28,29,30,31,32,33].

An alternative drug strategy against COVID-19 and related viral infectious diseases is the minimization of transmission [34,35]. It appears that insufficient efforts were introduced for preventing the transmission of the SARS-CoV-2 virus or for reducing the surrounding viral load or its nasopharyngeal viral entry [36]. Since according to Hippocrates, “prevention is better than treatment”, relevant strategies for reducing the transmission of the infection should be developed, including antivirals for preventing or reducing nasopharyngeal viral entry from flying droplets originating from the exhalation of infected SARS-CoV-2 individuals [36,37]. An important role in this strategy is the identification of infected areas, such as the development of a real-time environmental surveillance of SARS-CoV-2 aerosols [38].

While mRNA and other vaccines against the SARS-CoV-2 virus were considered the main front-line therapeutic approach for reducing the incidence of SARS-CoV-2 infection, repurposed antiviral, anti-inflammatory and other drugs also played a major role in the reduction in the associated mortality rate of infected patients. However, some questions remain on the efficacy of the available treatments. For example, it was estimated by the WHO that vaccines saved 0.5 million lives out of 5.0 million deaths by the end of 2021, while by 2023, the mortality rate reached almost 7.0 million people [1]. Furthermore, the SARS-CoV-2 virus has not yet been eliminated, and its long-term side effects affect millions of infected patients. For example, the current mortality rate in Europe due to COVID-19 is estimated at 1000 people every week, and in addition, about 1 in 30 Europeans have suffered from “long COVID” side effects in the last 3 years, including 17 million reported only for 2021 and 2022 [26,27,28,29,30,31,32,33].

Another major issue regarding future strategies and rapid interventions to stop pandemics in the future is the associated cost and other financial constraints [39]. This was one of the main reasons for the unavailability of diagnostics and therapeutics, not only in developing countries but also in developed countries, at the early stages of the COVID-19 pandemic. It is ironic that in 2023, the European Union destroyed unused vaccines against SARS-CoV-2, which is estimated to be worth EUR 4 billion. This happened because, despite the call for vaccinations, millions of Europeans decided not to follow warnings given by their health authorities. Investing in health as early as possible, especially for preventing pandemics such as COVID-19, may have been a wiser decision [39].

Overall, vaccinations against the SARS-CoV-2 virus can partly help reduce morbidity and mortality rates but are not the answer to eliminating or curbing the COVID-19 pandemic and its side effects, as well as future viral infections. In this context, there is a need for a long-term multilevel drug strategy targeting all aspects of viral infections, including the proliferation and associated life-threatening complications of SARS-CoV-2 and other viruses. One such life-threatening late complication in COVID-19 is sepsis, which is characterized by an unregulated host immune response to severe infection and mostly affects immunocompromised patients [11,40]. In this context, precision personalized medicine approaches are required, including the consideration of clinical, immunological, microbiological, pharmacological and other parameters [40,41]. Information from such data could lead to specific targeting antibacterial, antimicrobial and other drug combination therapeutic strategies, which based on appropriate algorithms and guidance from artificial intelligence techniques, could result in improved patient therapeutic outcomes [11,40,41]. The same precision personalized medicine strategy could also be adopted for future viral infections [11].

There is also a need for adopting the “one world one health”-model health system for the early identification, monitoring and therapeutic management of all aspects of pandemics, including therapeutic interventions as soon as possible [39,42,43]. This model for pandemics is necessary as it affects not only older populations and other susceptible groups but also the quality of life for all humans, as shown in the last few years by COVID-19 and previously by other similar viral infectious diseases.

## Figures and Tables

**Figure 1:**
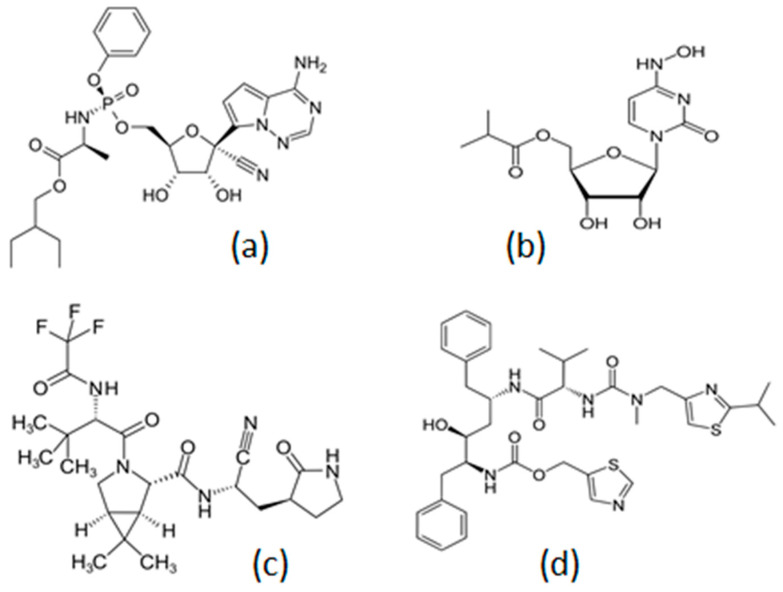
The chemical structure of the antiviral drugs (**a**) Remdesivir, (**b**) Molnupiravir, (**c**) Nirmatrelvir and (**d**) Ritonavir, which are widely used for the treatment of symptomatic SARS-CoV-2 virus-infected patients.
